# Mitochondrial health quality control: measurements and interpretation in the framework of predictive, preventive, and personalized medicine

**DOI:** 10.1007/s13167-022-00281-6

**Published:** 2022-05-12

**Authors:** Lenka Koklesova, Alena Mazurakova, Marek Samec, Erik Kudela, Kamil Biringer, Peter Kubatka, Olga Golubnitschaja

**Affiliations:** 1 Clinic of Obstetrics and Gynecology, Jessenius Faculty of Medicine, Comenius University in Bratislava, 036 01 Martin, Slovakia; 2 Department of Pathological Physiology, Jessenius Faculty of Medicine, Comenius University in Bratislava, 036 01 Martin, Slovakia; 3 Department of Medical Biology, Jessenius Faculty of Medicine, Comenius University in Bratislava, 036 01 Martin, Slovakia; 4 Predictive, Preventive, Personalised (3P) Medicine, Department of Radiation Oncology, University Hospital Bonn, Rheinische Friedrich-Wilhelms-Universität Bonn, 53127 Bonn, Germany

**Keywords:** Mitochondria, Health, Mitochondrial stress, Mitochondrial health index, Bioenergetic health index, Mitochondrial fusion and fission, Cell apoptosis, Systemic effects, Ischemia–reperfusion, Ischemic stroke, Disease development, Disease severity, COVID-19, Predictive preventive personalized medicine (PPPM/3PM), Primary secondary tertiary care, Health policy

## Abstract

Mitochondria are the “gatekeeper” in a wide range of cellular functions, signaling events, cell homeostasis, proliferation, and apoptosis. Consequently, mitochondrial injury is linked to systemic effects compromising multi-organ functionality. Although mitochondrial stress is common for many pathomechanisms, individual outcomes differ significantly comprising a spectrum of associated pathologies and their severity grade. Consequently, a highly ambitious task in the paradigm shift from reactive to predictive, preventive, and personalized medicine (PPPM/3PM) is to distinguish between individual disease predisposition and progression under circumstances, resulting in compromised mitochondrial health followed by mitigating measures tailored to the individualized patient profile. For the successful implementation of PPPM concepts, robust parameters are essential to quantify mitochondrial health sustainability. The current article analyses added value of Mitochondrial Health Index (MHI) and Bioenergetic Health Index (BHI) as potential systems to quantify mitochondrial health relevant for the disease development and its severity grade. Based on the pathomechanisms related to the compromised mitochondrial health and in the context of primary, secondary, and tertiary care, a broad spectrum of conditions can significantly benefit from robust quantification systems using MHI/BHI as a prototype to be further improved. Following health conditions can benefit from that: planned pregnancies (improved outcomes for mother and offspring health), suboptimal health conditions with reversible health damage, suboptimal life-style patterns and metabolic syndrome(s) predisposition, multi-factorial stress conditions, genotoxic environment, ischemic stroke of unclear aetiology, phenotypic predisposition to aggressive cancer subtypes, pathologies associated with premature aging and neuro/degeneration, acute infectious diseases such as COVID-19 pandemics, among others.

## Preamble

Mitochondrial health is crucial for myriads of physiologic process, continuity of molecular and sub/cellular functions performed in the human body. Intact mitochondrial functionality emerges as a key player in cell fate decisions coordinating cellular metabolism, immunity, and adequate stress response, among others. To exemplify some of the key points are the following:

- Mitochondria are the “gatekeeper” in a wide range of cellular functions, signaling events, cell homeostasis, proliferation, and apoptosis [1].
- Efficacy of DNA repair machinery and anti-aging protection [2] as well as healing capacity [3] are directly linked to the mitochondrial health.
- Systemic functionality (such as immune and cardio-vascular systems, digestive tract, reproductive health) [4–8], fetal development [9], and metal health [10] are all dependent on the level of mitochondrial network integrity and performance, mitochondrial health sustainability and quality control.

On the other hand, reduced health of the mitochondrial network is involved in pathomechanisms known as being related to compromised health conditions, such as progression from the reversible health damage (suboptimal health status) to clinically manifested disorders being, therefore, decisive for disease development, cause, and severity. This has been demonstrated for the majority of malignancies, cardio-vascular and neurological diseases, among others [11, 12].

Under imbalanced endogenous and/or exogenous stress conditions, such as heavy metal exposure [13] and depending on the severity of the mitochondrial stress, a vicious circle can be triggered by excessive ROS release and concomitant damage to mtDNA leading to insufficient energy production and uncontrolled increase in ROS release [12]. When the repair machinery fails, stressed cells undergo apoptosis by mitochondrial involvement: excessive fission of mitochondria and apoptotic cell death occur almost simultaneously [14]. Whereas mitochondrial fusion presents a compensatory mechanism which allows for maintaining sufficient energy output under adaptable stress conditions, when a tolerable threshold of damage is crossed, severely damaged mitochondria are eliminated (autophagy) from mitochondrial population by fission to preserve the health of the functional network. This mechanism provides measurable parameters for predictive diagnostics (such as extensive fission as the measure of imbalanced stress which cannot be compensated anymore) and valuable targets for effective prevention and personalized treatments.

This can be exemplified by the role of mitochondria in ischemic stroke (IS) prediction, prevention, and treatment. To this end, stroke is the leading cause of physical and intellectual disability in adults globally (currently over 60 million disability-adjusted life years) and major cause of mortality in developed countries [15]. Only a highly restricted portion of the patient cohort falls into a timely thrombolytic therapy/endovascular treatment window, which is extremely narrow. Since most acute IS patients currently receive no active treatment, innovative targeted therapy is requested. Mitochondria are a major target in hypoxic/ischemic injury [16]. Consequently, the mitochondrial health quality control is an ideal target for both—the IS risk assessment and neural protection, survival, and improved individual IS outcomes [15, 17, 18]. Moreover, mitochondrial health quality control may be of particular clinical utility in the case of cerebral small vessel disease giving rise to one in five stroke cases being a leading cause of cognitive impairment and dementia [19]. To this end, blood–brain barrier breakdown in the peri-infarct zone leading to secondary injury is linked to both—a limited recovery and significant alterations in mitochondrial health quality [20].

What are the parameters of the mitochondrial health index and how to correctly interpret research data available to predict mitochondrial health reduction–related disease development and severity grade followed by targeted prevention and treatment algorithms tailored to the individualized patient profile? The article does not pretend to answer all the questions. Herewith, we exemplify plausible pathways aimed at objective quantification of mitochondrial heath and do propose solutions for clinically relevant improvements in the framework of 3P medicine.

## Mitochondrial health index

**Fig. 1:**
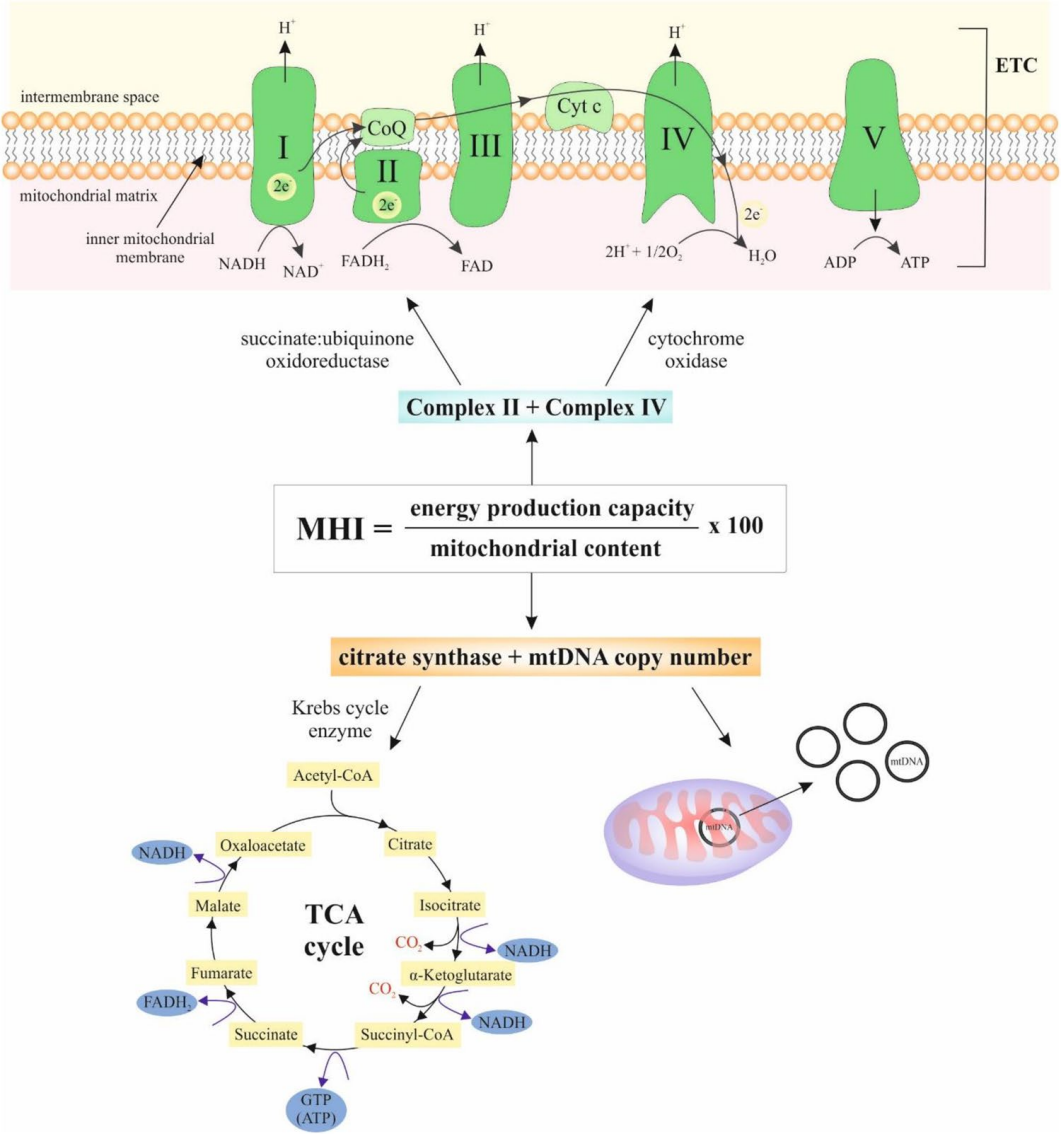
Formula for MHI calculation. Enzymes occurring in ETC: NADH–coenzyme Q reductase (complex I), succinate–coenzyme Q reductase/succinate dehydrogenase (complex II), coenzyme QH2 cytochrome-c reductase (complex III), cytochrome-c oxidase (complex IV), and ATP synthase (complex V). Enzymes occurring in TCA cycle: citrate synthase, aconitase, isocitrate dehydrogenase, α-ketoglutarate, succinyl-CoA synthetase, succinate dehydrogenase, fumarase, and malate dehydrogenase. *Abbreviations***:** mtDNA, mitochondrial DNA; TCA, tricarboxylic acid; NADH, nicotinamide adenine dinucleotide; FADH_2_, flavin adenine dinukleotide; CO_2_, carbon dioxide; Cyt c, cytochrome c; CoQ, coenzyme Q; ADP, adenosine diphosphate; ATP, adenosine triphosphate; GTP, guanosine triphosphate; ETC, electron transport chain; CoA, coenzyme A

Mitochondrial DNA (mtDNA) consists of 16,569 base pairs that encode 37 genes in human cells [21]. The mtDNA is important for normal mitochondrial function whereas encodes components of the respiratory chain complexes [22]. Mitochondria provide the energy (ATP) for cellular processes and generate adaptative signals, thus affecting the physiological response of the cells to various stressors [23, 24]. Although the human mtDNA is present in thousands of copies per cell, the mutations can affect only a part of them [25]. In the case of mitochondrial dysfunction, the number of copies of mtDNA per cell can be higher which is thought to be a compensation mechanism for poor mitochondrial quality. On the other hand, a decline in mtDNA copy number is more dramatic in older individuals [26]. Due to changes with time of day, aging, and disease, the mtDNA copy number alone is not a good marker of mitochondrial content or quality [27, 28]. MHI mathematically integrates mtDNA copy number and nuclear and mitochondrial DNA-encoded respiratory chain enzymatic activities into a single score with predictive potential. MHI could be determined by the following formula: MHI = (energy production capacity/mitochondrial content) × 100 (Fig. 1) [29].

### Alterations in mtDNA copy number

Due to the constant process of mitochondrial dynamics (fusion and fission), it is difficult to determine the accurate number of mtDNA molecules per mitochondrion [30]; however, a recent study revealed that energy-intensive tissues (cardiac and skeletal muscle) contained between 4000 and 6000 mtDNA copies per cell but liver, kidney, and lung tissues contained between 500 and 2000 mtDNA copies [31]. The level of mtDNA copy number can be measured from extracted DNA from peripheral blood or other tissues [32]. For achieving a relative measure of mtDNA copy number, the number of copies of a mitochondrial gene (*MT-ND1*, *MT-ND4*, *MT-CYB*, and *MT-TL1*) is usually compared to the number of copies of a nuclear gene (*B2M*, *RPLPO*, *ACTB*, and *RPPH1*) [33].

Alterations in mtDNA content are commonly related to various age-related diseases such as cardiovascular disease, type 2 diabetes, dementia, or cancer [34]. These alterations occur due to pathophysiological changes during the transition from healthy to diseased states [35]. From a molecular point of view, the changes can be associated with heterozygous disruption of human transcription factor A of mitochondria (*TFAM*) that leads to the decline in mtDNA copy number [25]. *TFAM* is necessary for mtDNA transcription initiation and essential for packaging mtDNA into mitochondrial nucleoids [36, 37]. Moreover, mtDNA copy number reduction is also related to a decline in mitochondrial transcription and decreased protein levels involved in OXPHOS such as ND1, CYTB, and COX-1 [38]. Copy number changes and point mutations are also the two most common mtDNA alterations in many human cancer types. Moreover, chemical depletion of mtDNA or impairment of mitochondrial respiratory chain in tumor cells promotes cancer progression to invasive phenotype or chemoresistance [39]. For example, a decline in mtDNA copy number in tumor tissue relative to adjacent normal tissue was determined in bladder, breast, esophageal, head and neck squamous cell, kidney, and liver cancers. Only lung adenocarcinomas revealed an increase in mtDNA copy number content [39]. Different results in the abundance of mtDNA copy numbers could be associated with different bioenergetic requirements of tumors or due to specific nuclear DNA or mtDNA mutation. For example, mtDNA content is significantly upregulated in endometrial carcinomas with *TP53* mutations or in low-grade gliomas caused by *PTEN* or *IDH1* mutations when compared to wild-type samples [40].

### Alterations in mitochondrial enzymatic activities

Circulatory or hormonal disturbances, poisoning, malnutrition, viral infection, or an extramitochondrial error of metabolism often correlates with mitochondrial enzyme deficiencies [41]. Moreover, the mitochondrial network has a special role in the metabolic-epigenome-genome axis due to the regulation of the level of key metabolites (acetyl CoA, NAD( +), and ATP) that act as the substrates or cofactors for kinases (protein kinase A), acetyl transferases, and deacetylases (sirtuins) [42]. Moreover, heterodimer mtDNA polymerase γ, mitochondrial single-strand DNA-binding protein (mtSSB), DNA helicase Twinkle, topoisomerase 3α, mitochondrial RNA polymerase (POLRMT), and DNA ligase III are the enzymes critical for mtDNA replication in vitro [43]; however, their mutations represent a major cause of various diseases [44, 45]. Moreover, below-described alterations of bioenergetic pathways, which include several mitochondrial enzymes, can be associated with changes in mtDNA copy number [46].

## Bioenergetic health index

**Fig. 2:**
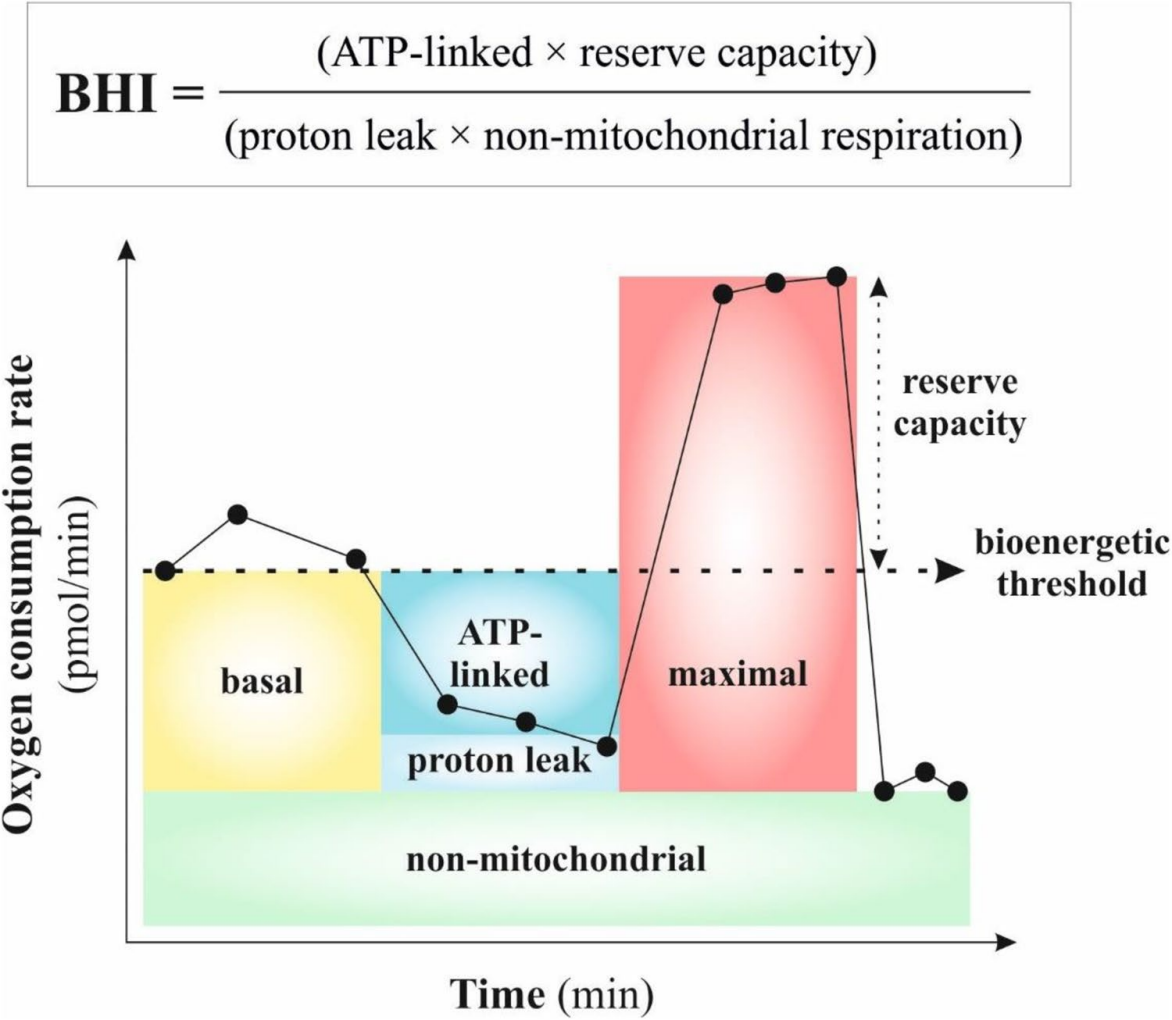
Formula for BHI calculation. These results can be detected in mitochondrial stress test by using of various modulators affecting the mitochondrial respiration: oligomycin (complex V inhibitor), carbonyl cyanide-4 (trifluoromethoxy) phenylhydrazone (FCCP) (oxygen consumption by complex IV reaches the maximum), and mixture of rotenone (complex I inhibitor) and antimycin A (complex III inhibitor) [52]

The bioenergetic pathways of mitochondria include ATP synthesis through OXPHOS, metabolite oxidation through the Krebs cycle, and β-oxidation of fatty acids [47]; however, various mitochondrial diseases caused by the mitochondrial dysfunction are most often related to OXPHOS defects [48]. OXPHOS dysfunction occurs in the case when mtDNA mutations exceed the critical threshold but it also depends on the type of mtDNA mutation and affected tissue [49]. Subsequently, bioenergetic and metabolic dysfunction lead to oxidative stress typical for many chronic diseases, including neurodegeneration, atherosclerosis, and diabetes [50]. BHI is a value representing the bioenergetic health of individuals [50]. Composite BHI represents an average ratio of OXPHOS to glycolysis. In mitochondrial health, the BHI is high that is associated with a high bioenergetic reserve capacity, high ATP-linked respiration, and low proton leak [51]. Moreover, BHI could be calculated by the following formula: BHI = (ATP-linked × reserve capacity)/(proton leak × non-mitochondrial) (Fig. 2). Therefore, BHI can represent a key concept that can be used against the bioenergetic crisis in patient populations [51].

### Alterations in bioenergetic pathways

Bioenergetic pathways are commonly based on an electron transport chain (ETC). The mitochondrial ETC consists of five enzyme complexes: NADH–coenzyme Q reductase (complex I), succinate–coenzyme Q reductase/succinate dehydrogenase (complex II), coenzyme QH2 cytochrome-c reductase (complex III), cytochrome-c oxidase (complex IV), and ATP synthase (complex V) [46]. The role of these enzymes is the transport from a donor (e.g., nicotinamide adenine dinucleotide (NADH)) to an electron acceptor (oxygen) [53]. The production of electron donors supplying ETC is regulated by many metabolic pathways, including the Krebs cycle, glycolysis, or β-oxidation [54]. Moreover, mitochondrial energy metabolism includes ATP generation (OXPHOS). The respiratory chain produces ATP from adenosine diphosphate (ADP) and inorganic phosphate through obtained energy from electrons (transferred from NADH or flavin adenine dinucleotide).

The evaluation of cellular bioenergetic profiles in health and disease is important to consider the key parameters of basal respiration, ATP-linked oxygen consumption, proton leak, and reserve capacity. During normal/unstressed conditions, the cells use only a fraction of their mitochondrial bioenergetic capacity known as spare or reserve respiratory capacity that is defined by a difference between the maximum respiratory capacity and basal respiratory capacity. In case of various diseases, when energy demand exceeds supply, the reserve respiratory capacity has the ability to increase supply to avoid an ATP crisis. Therefore, higher reserve capacity correlates with increased cell survival; however, decreased reserve capacity is associated with cell death and disease [55]. Furthermore, OXPHOS is powered by the proton gradient formed across the inner mitochondrial membrane and subsequently coupling respiratory oxygen to ADP phosphorylation/ATP generation [56]. In the absence of ADP, mitochondria are in state 2 or 4 respiration that is characterized by consumption of oxygen and a proton leak [57]. Furthermore, proton leak is also defined by the migration of protons to the matrix but independently of ATP synthase [58].

Alterations in bioenergetic pathways are related to the development of various heterogeneous metabolic diseases, including neurodegenerative disorders, cardiovascular or haematologic diseases, cancer, nephropathy, and diabetes [46, 59]. Defects in bioenergetic pathways, especially in ETC, are associated with energy imbalance, reactive oxygen species (ROS) production, disturbances in the redox state, mitochondrial membrane potential, mitochondrial protein import, apoptosis, or other signaling [53]. Furthermore, defects of complexes I, III, IV, and V are related to deficiency of mtDNA replication, RNA metabolism, or translation [46]. Moreover, several mitochondrial enzymes that are related to the mitochondrial tricarboxylic acid (TCA) cycle (also known as the Krebs cycle) are crucial for epigenetic remodeling. These enzymes are partially localized to the nucleus. Alterations of TCA cycle enzymes in entering the nucleus lead to the loss of specific histone modifications [60]. Additionally, mitochondrial dysfunction is also characterized by a loss of efficiency in OXPHOS and results in insufficient energy production for cells leading to an accumulation of ROS. Subsequently, ROS can damage lipids, proteins, and nucleic acids [61].

In the case of many types of cancer, due to the metabolic transformation of tumor tissues, molecular pathways often switch from OXPHOS to aerobic glycolysis. This phenomenon is also known as the Warburg effect. Aerobic glycolysis is important for cancer cells due to the compensation of efficiency of ATP production afforded by glycolysis when compared to mitochondrial OXPHOS. For this compensatory effect, the cancer cells upregulate glucose transporters (e.g. GLUT1, GLUT 3, aldolase-B, and hexokinase II). Moreover, higher levels of glycolytic enzymes such as hexokinase II, phosphofructokinase, phosphoglycerate kinase, and lactate dehydrogenase were observed in hypoxic tumor cells [62, 63].

## MHI and BHI as parameters to quantify mitochondrial health in disease development and severity

**Fig. 3:**
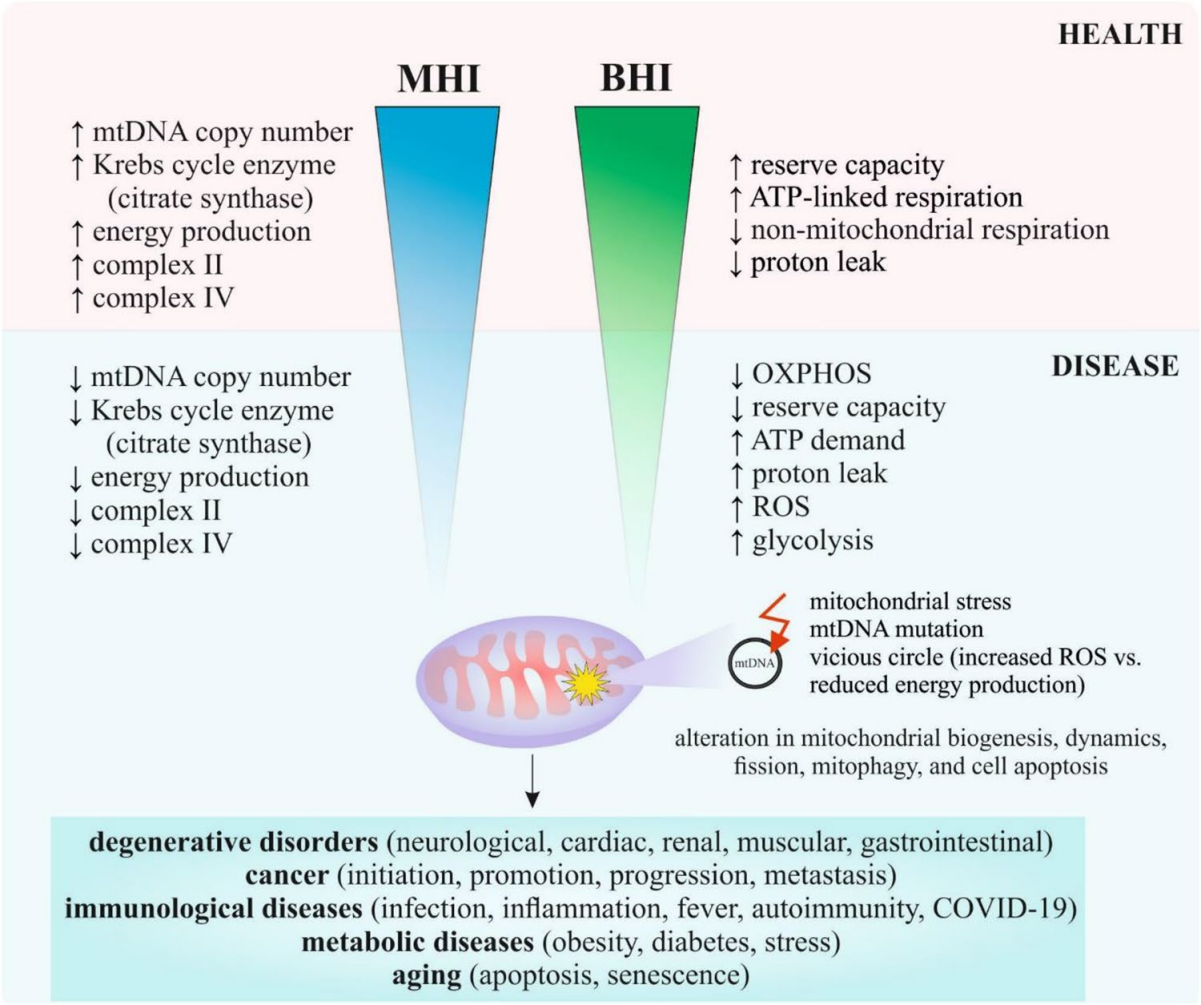
MHI and BHI in health and disease. *Abbreviations: *mtDNA, mitochondrial DNA; MHI, mitochondrial health index; BHI, bioenergetic health index; OXPHOS, oxidative phosphorylation; ATP, adenosine triphosphate

Several studies described the alterations only in mtDNA copy number in health and disease [64–69]; however, mtDNA alone is not enough indicative for the mitochondrial health evaluation. It is important to consider additional parameters such as mitochondrial enzymatic activities or modulations in bioenergetic pathways. BHI and MHI can serve as potential biomarkers for various disease development or severity. Whereas MHI and BHI represent a novel concept that mathematically integrates these several aspects into a single core, only limited studies have been described (Table 1). Figure 3 represents the MHI or BHI in health and disease.

**Table 1:** Altered MHI or BHI in clinically relevant pathologic conditions

Disease	Study design	Altered MHI or BHI	Results	Reference
Cardiac surgery	Healthy adult donors (*n* = 13) and adult patients (*n* = 14) undergoing cardiac surgery for ischaemic heart disease or valvular heart disease; primary adult rat cardiac myocytes	↓ BHI	↓ basal, ATP-linked, proton leak, ↓ maximal mitochondrial oxygen consumption rate, ↓ reserve capacity, ↓ mitochondrial membrane potential, ↑ ROS	[70]
Prostate cancer	RWPE-1, WPE1-NA22, WPE1-NB14, WPE1-NB11 and WPE1-NB26 cell lines	↓ MOBI	Invasive prostate cancer cells: ↓ OXPHOS, ↑ glycolysis, ↓ reserve capacity, ↑ oxygen consumption rate, ↓ extracellular acidification rate	[71]
Diabetic nephropathy	Peripheral blood mononuclear cells from healthy controls (*n* = 39), diabetic controls (*n* = 45), and diabetic nephropathy patients (*n* = 83)	↓ BHI	↓ reserve capacity, ↓ maximal respiration, ↓ metabolic flexibility	[72]
Caregiving stress	Peripheral blood mononuclear cells from healthy mothers of a child with an autism spectrum disorder (high-stress caregivers, *n* = 46) with mothers of a neurotypical child (control group, *n* = 45)	↓ MHI	↑ perceived stress, ↓ positive mood, ↑ negative daily affect, ↓ mitochondrial content of citrate synthase and mtDNA copy number, followed succinate dehydrogenase and COX	[29]

**Abbreviations:** ↑ = increased; ↓ = decreased

*MHI*, mitochondrial health index; *BHI*, bioenergetic health index; *MOBI*, mitochondrial oncobioenergetic index; *mtDNA*, mitochondrial DNA; *ROS*, reactive oxygen species; *ATP*, adenosine triphosphate; *COX*, cyclooxygenase

### Cardiovascular diseases

Different alterations in BHI can be obtained depending on the sample collection (blood or pericardial fluid) in the patient after cardiac surgery. Nevertheless, cardiac surgery is a common procedure that also causes many post-operative complications related to increased morbidity. When compared with healthy controls and blood samples, decreased BHI was obtained from the monocytes isolated from post-operative pericardial fluid that was characterized by a rapid decrease in basal, ATP-linked, proton leak, maximal mitochondrial oxygen consumption rate, and reserve capacity. Moreover, in vivo results revealed that post-operative pericardial fluid can act as a pro-oxidant through the loss of mitochondrial membrane potential and higher ROS production in rat cardiomyocytes [70].

### Cancer

In a prostate cancer study, the altered bioenergetic pathways were determined by a mitochondrial oncobioenergetic index (MOBI) that mathematically integrates oncobioenergetic profile of a cancer cell. MOBI significantly increases upon transformation into premalignant form and rapidly decreased in aggressive tumorigenesis. In prostate cancer cells, with increasing invasiveness (RWPE-1 < WPE-NA22 < WPE-NB14 < WPE-NB11 < WPE-NB26), the MOBI decreased. Early and late pre-malignant non-invasive cells demonstrated higher reserve capacity compared to RWPE-1 cells. On the contrary, early and late invasive WEP1-NB11 and WEP1-NB26 cell lines absolutely lacked reserve capacity. Moreover, during the transformation of normal cells into premalignant cells, the OXPHOS rapidly increased; however, in aggressive prostate cancer phenotype (after transformation), OXPHOS dramatically decreased. Eventually, during the transformation, glycolysis declined but after transformation, the glycolysis was elevated. As a result, bioenergetically, each cell line could be divided into energetic (RWPE-1), aerobic (WPE1-NA22, WPE1-NB14, and WPE1-NB11), and glycolytic (WPE1-NB26) phenotype. In conclusion, the energetics in premalignant WPE1-NA22 and WPE1-NB14 and early invasive WPE1-NB11 cells shifted to aerobic (Warburg) phenotype characterized by a high oxygen consumption rate and low extracellular acidification rate due to glycolysis [71].

### Renal diseases

Diabetic nephropathy affects approximately one-third of patients with diabetes that progress to end-stage renal failure despite therapy. Patients with diabetes had increased circulating mtDNA when compared with healthy individuals; however, in peripheral blood mononuclear cells, from diabetic nephropathy patients, reduced BHI was related to reduced reserve capacity and maximal respiration and loss of metabolic flexibility when compared with patients with diabetes [72].

### Mood and caregiving stress

Chronic caregiving stress and also daily mood are related to mitochondrial functional capacity. That is not new that chronic stress can cause behavioral, affective, cognitive, or metabolic changes leading to aging and disease predisposition. Some studies have found a decrease in mtDNA copy number, especially in posttraumatic stress disorder, depression, and in the elderly [26, 73, 74]. Another study revealed that higher MHI was observed in mothers of a neurotypical child that had a positive mood at night. On the other hand, higher perceived stress and lower positive and greater negative daily affect led to decreased MHI in healthy mothers of a child with an autism spectrum disorder. From enzymatical activity, the level of citrate synthase, COX, and succinate dehydrogenase was measured. Mitochondrial content of citrate synthase and mtDNA copy number were weakest, followed by nuclear-encoded succinate dehydrogenase and mtDNA encoded COX [29].

## Proposed MHI or BHI alterations

Despite the lack of evidence about MHI or BHI, there are several preclinical and clinical studies from which MHI or BHI could be evaluated but more detailed information is needed.

### Cardiovascular diseases

In the mice model of cardiomyopathy, mice carrying the heteroplasmic m.5024C > T mutation in the mitochondrial tRNA alanine (tRNA^Ala^) gene (C5024T mice) have many features of mild progressive cardiomyopathy and cytochrome *c* oxidase (COX) deficiency in many organs. The heteroplasmic C5024T mutation of the tRNA^Ala^ gene led to impaired mitochondrial translation. MtDNA copy number increased in C5024T mice at advanced disease stages, likely as a compensatory response induced by the mitochondrial dysfunction. In older mice (at 50 weeks of age), the mtDNA copy number increased, especially with high mutation levels in the colon tissue but not in heart tissue when compared with younger mice (at 20 weeks of age). Further analysis revealed that TFAM protein levels obtained from total tissue (colon and heart) extracts increased by ~ 50% in the overexpressing *TFAM* C5024T mice; however, the levels decreased by ~ 50% in the heterozygous *TFAM* knockout C5024T mice. In summary, increased *TFAM* expression and mtDNA copy number in mice with both wild-type and C5024T mutation mtDNA led to mild progressive cardiomyopathy and COX deficiency [75]. On the contrary, mtDNA copy number commonly decreases in heart failure. The association between mtDNA copy number and TFAM or Twinkle helicase in volume overload was described in an animal study of heart failure. Study with transgenic mice, one overexpressing TFAM and one overexpressing Twinkle helicase revealed that TFAM or Twinkle overexpression increased mtDNA copy number leading to facilitated cardioprotection and reduced mitochondrial oxidative stress [76].

Moreover, increased apoptotic signaling, redox stress, mitochondrial respiration defects, abnormal mitochondrial permeability transition pore opening, and failed antioxidant response were observed in induced pluripotent stem cell–derived cardiomyocytes (iPSC-CM) from patients with hypoplastic left heart syndrome with early heart failure when compared with iPSC-CM from patients without early heart failure. The study also revealed that uncompensated oxidative stress underlined early heart failure in hypoplastic left heart syndrome [77]. Apoptosis is also involved in the progression of heart failure. Mitochondrial dysfunction of cardiomyocytes caused the dysregulation of cellular energy metabolism and increased endothelin-1 expression followed by apoptosis induction (activated caspase-3 but no caspase-8). Moreover, the mitochondrial inhibitors (rotenone, cobalt chloride, and antimycin A) inhibited mitochondrial function at different sites of the ETC in cardiomyocytes and caused increased glucose consumption as a result of the switch from beta-oxidation of fatty acid to glycolysis [78]. Furthermore, non-surgical bleeding is a clinical complication in heart failure patients. Higher ROS production, oxidized low-density lipoproteins, Bax, and cytochrome c release, and decreased total antioxidant capacity and pro-survival proteins (Bcl-2, Bcl-xL) were detected in the bleeder group when compared with the non-bleeder group. These biomarkers of oxidative stress, evaluation of pro-survivals and pro-apoptotic proteins in platelets, mitochondrial damage, caspase activation, and platelet apoptosis could be used to identification of heart failure patients at high risk of non-surgical bleeding post-implant [79]. Moreover, increased expression of natriuretic peptide A and decreased transcripts of genes of cell survival and extracellular matrix were detected in patients with chronic primary mitral regurgitation. Decompensated chronic primary mitral regurgitation was related to decreased expression of *SERCA2*, *JUN*, *MAPK1*, and *MAPK8*, mitochondrial gene expression levels (*ATP5A1* and *PRDX3*), increased expression of genes associated with apoptosis (*FAS*, *PDCD1,* caspase-1, sarcolipin—SERCA regulatory protein, and chemokine (CxC motif) ligand 7), and inflammation when compared with compensated chronic primary mitral regurgitation. Moreover, calcium dysregulation and lower expression of genes important for bioenergetics were observed in patients with decompensated chronic primary mitral regurgitation [80].

### Ischemia–reperfusion injury

In the mice model of ischemia–reperfusion injury, a serine/threonine-protein phosphatase known as phosphoglycerate mutase 5 (PGAM5) localized in the outer mitochondria membrane can induce necroptosis depending on mitochondrial quality. In primary cardiomyocytes, the upregulation of PGAM5 induced cardiomyocyte necroptosis rather than apoptosis after ischemia–reperfusion injury. In this case, mtDNA copy number decreased that correlated with a decrease in state-3/4 respiration. On the other hand, PGAM5 deficiency/deletion led to increased mtDNA copy number and transcript levels, improved respiratory capacity, decreased mtROS production, and prevented abnormal mitochondrial permeability transition pore (mPTP) opening after ischemia–reperfusion injury. Moreover, cardiac-specific PGAM5 deletion attenuated cardiac inflammation and reduced myocardial infarction area [81]. Moreover, as a result of ischemia–reperfusion in the heart, excessive ROS, Bcl-2 proteins, and dysregulated calcium promoted mitochondrial membrane permeabilization resulting in the pro-apoptotic signaling through the release of cytochrome c and the high-temperature requirement serine peptidase 2 (HtrA2) into the cytosol. HtrA2 rapidly increased in ST-segment elevation myocardial infarction (STEMI) patients when compared with healthy controls. However, mitochondria-targeting peptide elamipretide reduced HtrA2 in STEMI patients through the retention of mitochondria by interaction with cardiolipin on the inner mitochondrial membrane. Therefore, the detection of HtrA2 in serum could be used as a biomarker for mitochondrial-induced cardiomyocyte apoptosis in patients with STEMI [82]. Moreover, the myocardium is exposed to ischemia–reperfusion injury during open-heart surgery. A pilot study demonstrated that treadmill exercise decreased mitochondrial respiration, cardiac troponin T, and triggered apoptosis through increased caspase-3 in the left ventricular after surgery. In conclusion, exercise could lead to increased susceptibility to perioperative damage to the myocardium and mitochondria [83]. Another study revealed that expression of miR-205 increased infarct size, oxidative stress, mitochondrial dysfunction, and apoptosis in mice with cardiac ischemia–reperfusion injury. On the other hand, inhibition of miR-205 decreased infarct size, mitigated apoptosis, oxidative stress increase, and mitochondrial fragmentation. MiR-205 inhibition was also associated with improved mitochondrial functional capacity and cardiac function [84].

Overexpression of RTN1-C and endoplasmic reticulum-associated protein localized in endoplasmic reticulum membrane mediated cerebral ischemia–reperfusion injury. This injury was associated with endoplasmic reticulum stress and mitochondria-associated apoptosis. RTN1-C increased cytosolic Bcl-xL and reduced mitochondrial Bcl-xL. On the other hand, knockdown of Rtn1-c inhibited apoptosis and the extent of ischemia–reperfusion-induced brain injury in middle cerebral artery occlusion stroke rats [85].

### Cancer

H^+^-ATP synthase acts as an engine of the inner mitochondrial membrane important for ATP synthesis by OXPHOS. Decreased expression of the β-catalytic subunit of the H^+^-ATP synthase is associated with an altered bioenergetic function of mitochondria in many types of cancer that is connected to the Warburg hypothesis. In most tumor samples (primary breast ductal invasive adenocarcinomas, gastric cancer, squamous lung or oesophageal carcinomas) decreased OXPHOS markers (β-F1-ATPase, heat-shock protein 60 (hsp60)) but increased glycolytic markers (glyceraldehyde-3-phosphate dehydrogenase (GAPDH) and pyruvate kinase) were observed when compared with the normal breast, gastric, lung, or oeasophageal tissues. On the other hand, changes in OXPHOS or glycolytic markers were not altered in prostate adenocarcinomas [86].

Although thermotherapy can be successfully used as a therapeutic strategy for various cancers, a higher temperature can affect cancer bioenergetics (multiple components of glycolytic and mitochondrial function). In SW480 and Pt.93 colon cancer cells, a higher temperature (42 °C) increased the proton leak when compared with basal temperature (37 °C); however, other components of oxygen consumption rate remained unchanged. On the other hand, more affected bioenergetics was observed in glycolysis when compared with mitochondrial respiration components. Hyperthermia increased all components of glycolysis, including non-glycolytic acidification, glycolysis, glycolytic capacity, and glycolytic reserve. Therefore, it is important to consider also bioenergetic pathways (glycolysis and OXPHOS) in cancer treatment by hyperthermia due to various changes [87].

### COVID-19

Coronavirus disease 2019 (COVID-19), an infectious disease caused by coronavirus-2 (SARS-CoV-2), causes various serious complications such as acute respiratory syndrome, a characteristic hyperinflammatory response, vascular damage, microangiopathy, angiogenesis, and widespread thrombosis [88]. Moreover, COVID-19 is also characterized by altered bioenergetics. Monocytes play an important role in the SARS‐CoV‐2 infection that often leads to inflammation and pneumonia. Altered bioenergetics was found in peripheral blood monocytes from patients with COVID‐19 pneumonia compared to healthy individuals. This state was associated with reduced basal and maximal respiration, spare respiratory capacity, and proton leak. Furthermore, many monocytes had abnormal mitochondrial ultrastructure and depolarized mitochondria. In summary, the infection with SARS-CoV-2 could significantly affect the monocytic compartment of innate immunity [89].

### Neurodegenerative diseases

Alzheimer’s disease, the most known age-related neurodegenerative disease, is characterized by various metabolic deficits, including glycolysis dysfunction, glucose metabolism dysregulation, TCA cycle dysregulation, OXPHOS deficits, or pentose phosphate pathway impairment [90]. Altered bioenergetic pathways through increased ROS production and reduced glycolysis and mitochondrial oxygen consumption were observed in immortalized hippocampal astrocytes from 3xTg-AD mice. Furthermore, the expression of mitochondrial and OXPHOS proteins did not modulate in mitochondria-endoplasmic reticulum-enriched fraction; however, the endoplasmic reticulum functions, Ca^2+^ homeostasis, and protein synthesis were deregulated [91]. Moreover, mitochondrial dysfunction can be associated with epigenetic changes. MtDNA methylation can contribute to the development of neurodegenerative diseases such as Alzheimer’s disease. The study of Xu et al. revealed that epigenetic hypermethylation of mitochondrial cytochrome b (*CYTB*) and *COX-2* genes decreased mtDNA copy numbers and expression in the hippocampi of APP/PS1 transgenic mice as a model of Alzheimer's disease. In conclusions, mtDNA methylation of *CYTB* and *COX-2* could lead to altered ETC enzymatical activities, especially in complex III and IV [92].

### Renal diseases

Hypertension has a causal role in the pathogenesis of kidney disease because worsens the clinical course of patients with chronic kidney disease that often leads to disease progression [93]. In a study by Eirin et al., the increased markers of renal injury and dysfunction in patients with hypertension were related to increased urinary mtDNA copy numbers of *COX-3* and *NADH dehydrogenase subunit-1* genes when compared with healthy volunteers. Higher urinary mtDNA copy number also correlated with urinary neutrophil gelatinase-associated lipocalin and kidney injury molecule-1 [94].

Patients with diabetic kidney disease, a tubular injury, had decreased mtDNA copy numbers and increased mtDNA damage compared to diabetic patients without kidney injury. Moreover, an accumulation of damaged mtDNA and fragmented mitochondria led to the bioenergetic (glycolysis and TCA cycle) alterations. Increased levels of dihydroxyacetone phosphate (glycolysis) and succinyl-CoA synthetase (TCA cycle) were obtained in diabetic kidney disease compared to healthy controls. Furthermore, patients with diabetic kidney disease had also increased ROS generation, activation of apoptosis, and loss of mitochondrial membrane potential in tubules and peripheral blood mononuclear cells [95].

### Gastrointestinal disorders

Impaired mitochondrial bioenergetics function was detected in pediatric chronic overlapping pain conditions patients with functional gastrointestinal disorders. Decreased ATP production by OXPHOS was associated with lower basal respiration and ATP-linked oxygen consumption and decreased glycolysis was related to a lower extracellular acidification rate when compared with healthy controls. A better predictor of functional disability in patients could be the spare respiratory capacity that rapidly increased with a greater disability [96].

### Obesity

Obesity represents a risk factor for chronic kidney disease. In obese patients, the urinary mtDNA copy number of nicotinamide adenine dinucleotide dehydrogenase subunit-1 increased when compared with healthy volunteers. On the contrary, urinary or serum mtDNA copy number of COX-3 and serum mtDNA copy numbers of nicotinamide adenine dinucleotide dehydrogenase subunit-1 did not change in the obese group compared to healthy volunteers. Moreover, bariatric surgery reduced the mtDNA copy number of nicotinamide adenine dinucleotide dehydrogenase subunit-1 in the high baseline mtDNA copy-number group. Results suggested that urinary mtDNA copy number, more specifically nicotinamide adenine dinucleotide dehydrogenase subunit-1, can be used as a potential marker of mitochondrial damage in various kidney diseases [97].

### Porphyria

Porphyria is a group of metabolic disorders associated with altered enzyme activities within the heme biosynthetic pathway. The bioenergetic profile of patients with porphyria revealed that oxygen consumption rate decreased through lower basal, ATP-linked, proton leak, maximal, reserve, and non-mitochondrial respiration when compared with healthy controls. Decreased oxygen consumption rate in porphyria patients is suggested to be caused by oxidative stress mediated by higher calcium cycling and subsequently by a decreased efficiency of the mitochondrial ATP generation [98].

### Rheumatoid arthritis

Rheumatoid arthritis is an autoimmune inflammatory disorder joint disease characterized by cartilage and bone damage, chronic pain and swelling, and disability [99]. Oxidative stress and mitochondrial alterations contribute to the pathogenesis of rheumatoid arthritis. Furthermore, mtDNA copy number is commonly significantly lower in patients with established rheumatoid arthritis. Study of Gautam et al. revealed that regular yoga practice in patients with rheumatoid arthritis could improve mitochondrial health. Significant increase in mtDNA copy numbers, transcripts that maintain mitochondrial integrity (5′ adenosine monophosphate-activated protein kinase (*AMPK*), tissue inhibitor of matrix metalloproteinases 1 (*TIMP-1*), a Greek word for the gene that regulates lifespan (*KLOTHO*), and *TFAM*), and mitochondrial activity markers (NAD + , COX-2, and mitochondrial membrane potential) after 8-weeks of yoga were detected in yoga group when compared with the non-yoga group. Moreover, the optimization of oxidative stress markers (lower ROS and higher total antioxidant capacity) and circadian rhythm markers (lower cortisol and higher melatonin, and higher serotonin) was also observed. In summary, regular yoga practice in rheumatoid arthritis patients could enhance mitochondrial health and reduce disease activity; therefore, it could be beneficial as an adjunct therapy [100].

### Oocyte’s vitrification

Women with infertility problems, usually visit the reproductive center in an effort to become pregnant. Mitochondrion has an important role in the production of energy for oocytes as an indicator of cytoplasmic maturation [101, 102]. A decrease in mitochondria of aged oocytes leads to lower fertilization rates and poor embryonic development [103]. In assisted reproductive technologies for fertility preservation, the mitochondria of oocytes could damage during the cryopreservation process; therefore, vitrification is a more suitable method because no ice crystals are formed. In vitrified oocytes obtained from super-ovulated adult female mice, mtDNA copy number, COX activity, and *TFAM* gene expression decreased but the ROS level increased in comparison with non-vitrified oocytes. Moreover, the tendency to succinate dehydrogenase decrease but was not statistically approved was detected in vitrified oocytes when compared with non-vitrified oocytes [104].

Several above-mentioned diseases are characterized by altered bioenergetics, nuclear or mitochondrial DNA-encoded enzymatical activities, or mtDNA copy numbers. In some studies, the MHI or BHI could be probably calculated. On the other hand, especially in MHI determination, some missing (mostly Krebs cycle enzyme activity) parameters would need to be supplemented. It is more difficult to understand MHI also due to differences in mtDNA copy numbers in health and disease because in some diseases mtDNA copy number increases and sometimes decreases. Therefore, more comprehensive studies are needed. Table 2 includes the overview of preclinical and clinical studies associated with altered mtDNA copy numbers, mitochondrial enzymatic activities, or bioenergetic pathways in many pathologies with the potential to determine MHI or BHI.

**Table 2:** Altered mtDNA copy numbers, mitochondrial enzymatic activities, and bioenergetic pathways in clinically relevant conditions

Disease	Study design	Results	Supposed MHI or BHI alteration	Reference
Cardiomyopathy	Colon and heart tissue from mice carrying the heteroplasmic m.5024C > T mutation in the mitochondrial tRNA alanine (tRNA^Ala^) gene (C5024T mice)	↑ mtDNA copy number, ↑ *TFAM*, ↑ COX deficiency	↓ MHI	[75]
Heart failure	C57BL/6 J transgenic mice overexpressing human TFAM or murine Twinkle	↑ TFAM, ↑ Twinkle helicase, ↑ mtDNA copy number, ↓ mitochondrial oxidative stress, ↑ cardioprotection	↑ MHI	[76]
Heart failure	Cultured cardiomyocytes	↑ apoptotis, ↑ caspase-3, ↑ heart failure progression, ↑ mitochondrial dysfunction, dysregulation of cellular energy metabolism, ↑ endothelin 1	↓ MHI	[78]
Non-surgical bleeding in heart failure	Multiple blood samples from patients with heart failure: bleeder (n = 12) and non-bleeder (n = 19) groups	↑ ROS, ↑ oxidative stress, ↑ oxidized low-density lipoproteins, ↑ apoptosis, ↑ Bax, ↑ cytochrome c release, ↓ total antioxidant capacity, ↓ Bcl-2, ↓ Bcl-xL,	↓ MHI	[79]
Hypoplastic left heart syndrome with early heart failure	Induced pluripotent stem cell–derived cardiomyocytes from patients with hypoplastic left heart syndrome with early heart failure	↑ apoptotic signaling, ↑ redox stress, ↑ uncompensated oxidative stress, ↑ mitochondrial respiration defects, ↑ mitochondrial permeability transition pore opening, ↓antioxidant response	↓ MHI	[77]
Decompensated chronic primary mitral regurgitation	Left ventricular endomyocardial biopsies (n = 12) from normal hearts (n = 5), patients with compensated (n = 6) and decompensated (n = 6) chronic primary mitral regurgitation	↑ natriuretic peptide A, ↑ cell survival genes, ↑ extracellular matrix genes, ↓ *SERCA2*, ↓ *JUN,* ↓ *MAPK1,* ↓ *MAPK8,* ↓ *ATP5A1,* ↓ *PRDX3, *↑ apoptosis, ↑ *FAS*, ↑ *PDCD1,* ↑ caspase-1, ↑ sarcolipin—SERCA regulatory protein, ↑ chemokine (CXC motif) ligand 7), ↑ inflammation, ↑ calcium dysregulation, ↓ expression of genes involved in bioenergetic pathways	↓ MHI, ↓ BHI	[80]
Cardiac ischemia–reperfusion injury	Cardiac-specific PGAM5 knockout (PGAM5CKO, PGAM5f/f, α-MHCCre +) mice	↑ PGAM5, ↓ mtDNA copy number, ↑ necroptosis, ↓ state-3/4 respiration	↓ MHI	[81]
ST-segment elevation myocardial infarction	Peripheral blood was obtained from patients (*n* = 19) with first-time acute anterior STEMI after percutaneous coronary intervention and healthy donors (*n* = 16)	↑ HtrA2, ↑ apoptosis	↓ MHI	[82]
Cardiac ischemia–reperfusion injury	Right atrial and left ventricular biopsies from patients scheduled for elective coronary artery bypass: treadmill exercise group (*n* = 10) 24 h preoperatively or standard presurgical procedures (*n* = 10)	↓ mitochondrial respiration, ↑ cardiac troponin T, ↑ apoptosis, ↑ caspase-3, ↑ perioperative damage of mitochondria and myocardium	↓ MHI	[83]
Cardiac ischemia–reperfusion injury	Male C57BL/6 mice with ischemia–reperfusion injury	↑ miR-205, ↑ infarct size, ↑ oxidative stress, ↑ mitochondrial dysfunction, ↑ apoptosis	↓ MHI	[84]
Ischemia–reperfusion-induced brain injury	Rat middle cerebral artery occlusion stroke and oxygen–glucose deprivation followed by reoxygenation model	↑ RTN1-C, ↑ endoplasmic reticulum stress, ↑ apoptosis, ↓ mitochondrial Bcl-xL, ↑ cytosolic Bcl-xL,	↓ MHI	[85]
Ductal invasive breast adenocarcinomas, gastric adenocarcinomas, and squamous oesophageal and lung carcinomas	Frozen tissue sections from human biopsies of untreated patients with primary ductal invasive breast adenocarcinomas, gastric and prostate adenocarcinomas, and squamous oesophageal and lung carcinomas	↓ OXPHOS markers: ↓ β-F1-ATPase, ↓ hsp60, ↑ glycolytic markers: ↑ GAPDH, ↑ pyruvate kinase	↓ BHI	[86]
Hyperthermia treatment for colon cancer	SW480 and Pt.93 colon cancer cells exposed to 32 °C, 37 °C and 42 °C for 60 min	↑ proton leak, ↑ non-glycolytic acidification, ↑ glycolysis, ↑ glycolytic capacity, ↑ glycolytic reserve	↓ BHI	[87]
COVID‐19 pneumonia	Peripheral blood monocytes from patients with COVID‐19 pneumonia (*n* = 28) and healthy controls (*n* = 27)	↓ basal and maximal respiration, ↓ spare respiratory capacity, ↓ proton leak, abnormal mitochondrial ultrastructure, depolarized mitochondria	↓ BHI	[89]
Alzheimer’s disease	Immortalized hippocampal astrocytes from 3xTg-AD mice	↑ ROS production, ↓ glycolysis, ↓ mitochondrial oxygen consumption, deregulations of endoplasmic reticulum functions, Ca^2+^ homeostasis, and protein synthesis	↓ BHI	[91]
	APP/PS1 transgenic mice	↑ methylation *CYTB,* ↑ methylation *COX-2,* ↓ mtDNA copy numbers	↓ MHI	[92]
Renal injury in human hypertension	Blood and urine samples from essential (*n* = 25) and renovascular (*n* = 34) hypertensive patients, and healthy volunteers (*n* = 22)	↑ copy numbers of mtDNA genes *COX-3* and *NADH dehydrogenase subunit-1*, ↑ urinary neutrophil gelatinase-associated lipocalin, ↑ kidney injury molecule-1	↓ MHI	[94]
Diabetic kidney disease	Serum, peripheral blood mononuclear cells and kidney biopsy specimens from healthy controls (*n* = 65), diabetes patients without kidney disease (*n* = 48) and diabetic kidney disease patients (*n* = 60)	↓ copy numbers of mtDNA, ↑ mtDNA damage, ↑ dihydroxyacetone phosphate, ↑ succinyl-CoA synthetase, ↑ ROS, ↑ apoptosis, ↓ mitochondrial membrane potential	↓ MHI	[95]
Pediatric chronic overlapping pain conditions with functional gastrointestinal disorders	Peripheral blood mononuclear cells from children aged 10–18 years with chronic overlapping pain conditions patients (*n* = 31) and 19 healthy controls (*n* = 19)	↓ OXPHOS, ↓ ATP production, ↓ basal respiration, ↓ ATP-linked oxygen consumption, ↓ glycolysis, ↓ extracellular acidification rate, ↑ spare respiratory capacity	↓ BHI	[96]
Obesity-associated kidney injury	Age- and sex-matched healthy volunteers (*n* = 22, 9 men and 13 women) and patients with obesity (*n* = 22, 9 men and 13 women)	↑ urinary mtDNA copy number of nicotinamide adenine dinucleotide dehydrogenase subunit-1, after bariatric surgery: ↓ urinary mtDNA copy number of nicotinamide adenine dinucleotide dehydrogenase subunit-1	↓ MHI	[97]
Porphyria	peripheral blood mononuclear cells from porphyria patients (*n* = 22) (12 porphyria cutanea tarda, 7 acute hepatic porphyria, and 3 erythropoietic protoporphyria) patients and age and gender-matched healthy controls (*n* = 18)	↓ oxygen consumption rate, ↓ basal, ↓ ATP-linked, ↓ proton leak, ↓ maximal, ↓ reserve, and ↓ non-mitochondrial respiration, ↑ oxidative stress, ↑ calcium cycling, ↓ mitochondrial ATP generation	↓ BHI	[98]
Yoga practice in patients with rheumatoid arthritis	Total participants with rheumatoid arthritis (*n* = 70)—yoga group (*n* = 35) and non-yoga group (*n* = 35); yoga group—standardized yoga practice five times a week for 120 min duration per session for 8-weeks	↑ mtDNA copy numbers, ↑ *AMPK*, ↑ *TIMP-1*, ↑ *KLOTHO*, ↑ *TFAM*, ↑ NAD + , ↑ COX-2, ↑ mitochondrial membrane potential, ↓ ROS, ↑ total antioxidant capacity, ↓ cortisol, ↑ melatonin, ↑ serotonin	↑ MHI	[100]
Oocyte’s vitrification	Collected metaphase II oocytes (*n* = 320) from super-ovulated adult female mice: randomly division into vitrified (*n* = 160) and non-vitrified (*n* = 160) groups	In vitrified oocytes: ↓ mtDNA copy number, ↓ COX, ↓ *TFAM,* tendency to succinate dehydrogenase decrease, ↑ ROS	↑ MHI	[104]

**Abbreviations:** ↑ = increased; ↓ = decreased

*MHI*, mitochondrial health index; *BHI*, bioenergetic health index; *TFAM*, human transcription factor A of mitochondria; *mtDNA*, mitochondrial DNA; *PGAM5*, phosphoglycerate mutase 5; *ROS*, reactive oxygen species; *ATP*, adenosine triphosphate; *hsp60*, heat-shock protein 60; *GAPDH*, glyceraldehyde-3-phosphate dehydrogenase; *CYTB,* mitochondrial cytochrome b; *COX*, cyclooxygenase; *COX-2*, cytochrome c oxidase 2; *COX-3*, cytochrome c oxidase 3; *Ca*^*2*+^, calcium cation; *AMPK,* 5′ adenosine monophosphate-activated protein kinase; *TIMP-1,* tissue inhibitor of matrix metalloproteinases 1; *KLOTHO,* a Greek word for the gene that regulates lifespan; NAD + , nicotinamide adenine dinucleotide; Bcl-2, B-cell lymphoma 2; Bcl-xL; B-cell lymphoma-extra large; Bax; BCL2 associated X; *SERCA2*, sarcoplasmic/endoplasmic reticulum calcium ATPase 2; *JUN,* Jun proto-oncogene, AP-1 transcription factor subunit; *MAPK,* mitogen-activated protein kinase*, ATP5A1,* mitochondrial membrane ATP synthase; *PRDX3,* peroxiredoxin 3; *FAS*, Fas cell surface death receptor; *PDCD1,* programmed cell death protein 1; *HtrA2*, HtrA serine peptidase 2; *RTN1-C*, reticulon protein 1-C; *miR-205*, micro RNA 205

## Conclusions and expert recommendations in the framework of 3P medicine

Mitochondria are the “gatekeeper” in a wide range of cellular functions, signaling events, cell homeostasis, proliferation, and apoptosis. Consequently, mitochondrial injury is linked to systemic effects compromising multi-organ functionality including but not restricted to the cardiac injury and heart failure, mental health (fatigue, dementia, ataxia, epilepsy, stroke, etc.), affected peripheral nervous and endocrine systems, bone marrow functionality, eyes (cataract, retinopathy, glaucomatous optic nerve degeneration, etc.) and ears (tinnitus and deafness) disorders, gut and kidney injury as well as compromised quality of reproductive functions at multiple levels [11]. To this end, “vicious circle” (uncontrolled ROS release, diminished energy production, and extensive oxidative stress to mtDNA and chrDNA) is characteristic for progressing reciprocal mitochondrial / multi-organ damage. Although mitochondrial stress is common for many pathomechanisms, individual outcomes differ significantly comprising a spectrum of associated pathologies and their severity grade [12]. Consequently, a highly ambitious task in the paradigm shift from reactive to predictive, preventive, and personalized medicine is to apply qualitative and quantitative analytic approaches, in order to distinguish between individual disease predisposition and progression under circumstances resulting in compromised mitochondrial health. For the successful implementation of PPPM concepts, robust parameters are essential to quantify mitochondrial health sustainability. The current article analyses added value of MHI and BHI as potential systems to quantify mitochondrial health relevant for the disease development and its severity grade. It is evident that more parameters are needed to cover the limitations of both systems, if applicable to primary care (reversible health damage, personalized protection against disease development), secondary care (disease prognosis, personalized treatments and protection against cascading complications) and tertiary care (stability of severe chronic pathologies).

General mitigating measures against oxidative mitochondrial damage are based on the antioxidant-defense with scavenging activities and individualized lifestyle recommendations including personally coached physical activities and dietary habits [11, 105]. Furthermore, detailed phenotyping was demonstrated as being instrumental for cost-effective screening programs dedicated to the needs of young populations in suboptimal health conditions such as vasospastic individuals strongly predisposed to systemic ischemia–reperfusion, mitochondrial injury and associated pathologies developed over the entire lifespan [106, 107].

The current article exemplified health conditions which MHI and BHI have been created for. Based on the pathomechanisms related to the compromised mitochondrial health and in the context of primary, secondary, and tertiary care, a broad spectrum of conditions can significantly benefit from robust quantification systems using MHI / BHI as a prototype to be further improved. Following health conditions can benefit from that:

- Planned pregnancies (improved outcomes for mother and offspring health)
- Suboptimal health conditions with reversible health damage
- Suboptimal life-style patterns and metabolic syndrome(s) predisposition
- Multi-factorial stress conditions
- Genotoxic environment
- Stroke of unclear aetiology
- Phenotypic predisposition to aggressive cancer subtypes
- Pathologies association with premature aging and neurodegeneration
- Acute infectious diseases such as COVID-19 pandemics among others.

## Data Availability

Not applicable.

## References

[1] Rath E, Moschetta A, Haller D (2018). Mitochondrial s. Nat Rev Gastroenterol Hepatol.

[2] Barja G (2021). Higher DNA Repair in Mitochondria of Long-Lived Species. Aging (Albany NY).

[3] Levoux J, Prola A, Lafuste P, Gervais M, Chevallier N, Koumaiha Z, Kefi K, Braud L, Schmitt A, Yacia A (2021). Platelets facilitate the wound-healing capability of mesenchymal stem cells by mitochondrial transfer and metabolic reprogramming. Cell Metab.

[4] Faas MM, de Vos P (2020). Mitochondrial function in immune cells in health and disease. Biochim Biophys Acta Mol Basis Dis.

[5] Camaioni A, Ucci MA, Campagnolo L, De Felici M, Klinger FG (2022). Italian Society of Embryology, Reproduction and Research (SIERR) The process of ovarian aging: it is not just about oocytes and granulosa cells. J Assist Reprod Genet.

[6] Rahimi A, Asadi F, Rezghi M, Kazemi S, Soorani F, Memariani Z (2022). Natural products against cisplatin-induced male reproductive toxicity: a comprehensive review. J Biochem Mol Toxicol.

[7] Ho G-T, Theiss AL (2022). Mitochondria and inflammatory bowel diseases: toward a stratified therapeutic intervention. Annu Rev Physiol.

[8] Ungvari Z, Tarantini S, Donato AJ, Galvan V, Csiszar A (2018). Mechanisms of vascular aging. Circ Res.

[9] Burton GJ, Jauniaux E (2018). Pathophysiology of placental-derived fetal growth restriction. Am J Obstet Gynecol.

[10] Zorov DB, Juhaszova M, Sollott SJ (2014). Mitochondrial reactive oxygen species (ROS) and ROS-induced ROS release. Physiol Rev.

[11] Liskova A, Samec M, Koklesova L, Kudela E, Kubatka P, Golubnitschaja O. Mitochondriopathies as a Clue to systemic disorders—analytical tools and mitigating measures in context of predictive, preventive, and personalized (3P) medicine. Int J Mol Sci. 2021;22. 10.3390/ijms22042007. 10.3390/ijms22042007 PMC7922866 33670490

[12] Koklesova L, Samec M, Liskova A, Zhai K, Büsselberg D, Giordano FA, Kubatka P, Golunitschaja O. Mitochondrial impairments in aetiopathology of multifactorial diseases: common origin but individual outcomes in context of 3P medicine. EPMA J. 2021;12:27–40. 10.1007/s13167-021-00237-2. 10.1007/s13167-021-00237-2 PMC7931170 33686350

[13] Sun Q, Li Y, Shi L, Hussain R, Mehmood K, Tang Z, Zhang H (2022). Heavy metals induced mitochondrial dysfunction in animals: molecular mechanism of toxicity. Toxicology.

[14] Youle RJ, van der Bliek AM (2012). Mitochondrial Fission, Fusion, and Stress. Science.

[15] Yang J-L, Mukda S, Chen S-D (2018). Diverse Roles of Mitochondria in Ischemic Stroke. Redox Biol.

[16] Ham PB, Raju R (2017). Mitochondrial function in hypoxic ischemic injury and influence of aging. Prog Neurobiol.

[17] Anzell AR, Maizy R, Przyklenk K, Sanderson TH (2018). Mitochondrial quality control and disease: insights into ischemia-reperfusion injury. Mol Neurobiol.

[18] He Z, Ning N, Zhou Q, Khoshnam SE, Farzaneh M (2020). Mitochondria as a therapeutic target for ischemic stroke. Free Radic Biol Med.

[19] Teng Z, Dong Y, Zhang D, An J, Lv P (2017). Cerebral small vessel disease and post-stroke cognitive impairment. Int J Neurosci.

[20] Nahirney PC, Reeson P, Brown CE (2016). Ultrastructural analysis of blood-brain barrier breakdown in the peri-infarct zone in young adult and aged mice. J Cereb Blood Flow Metab.

[21] Yan C, Duanmu X, Zeng L, Liu B, Song Z (2019). Mitochondrial DNA: distribution, mutations, and elimination. Cells.

[22] Nicholls DG, Ferguson SJ. Cellular bioenergetics. In: Bioenergetics 4th edition. Amsterdam: Academic Press; 2013.

[23] Picard M, McManus MJ, Gray JD, Nasca C, Moffat C, Kopinski PK, Seifert EL, McEwen BS, Wallace DC (2015). Mitochondrial functions modulate neuroendocrine, metabolic, inflammatory, and transcriptional responses to acute psychological stress. Proc Natl Acad Sci U S A.

[24] Chandel NS (2015). Evolution of mitochondria as signaling organelles. Cell Metab.

[25] Filograna R, Mennuni M, Alsina D, Larsson N-G (2021). Mitochondrial DNA copy number in human disease: the more the better?. FEBS Lett.

[26] Mengel-From J, Thinggaard M, Dalgård C, Kyvik KO, Christensen K, Christiansen L (2014). Mitochondrial DNA copy number in peripheral blood cells declines with age and is associated with general health among elderly. Hum Genet.

[27] Giordano C, Iommarini L, Giordano L, Maresca A, Pisano A, Valentino ML, Caporali L, Liguori R, Deceglie S, Roberti M (2014). Efficient mitochondrial biogenesis drives incomplete penetrance in Leber’s hereditary optic neuropathy. Brain.

[28] Yu-Wai-Man P, Sitarz KS, Samuels DC, Griffiths PG, Reeve AK, Bindoff LA, Horvath R, Chinnery PF (2010). OPA1 mutations cause cytochrome c oxidase deficiency due to loss of wild-type MtDNA molecules. Hum Mol Genet.

[29] Picard M, Prather AA, Puterman E, Cuillerier A, Coccia M, Aschbacher K, Burelle Y, Epel ES (2018). A Mitochondrial health index sensitive to mood and caregiving stress. Biol Psychiatry.

[30] Wai T, Ao A, Zhang X, Cyr D, Dufort D, Shoubridge EA (2010). The Role of Mitochondrial DNA Copy Number in Mammalian Fertility. Biol Reprod.

[31] D’Erchia AM, Atlante A, Gadaleta G, Pavesi G, Chiara M, De Virgilio C, Manzari C, Mastropasqua F, Prazzoli GM, Picardi E (2015). Tissue-specific MtDNA abundance from exome data and its correlation with mitochondrial transcription, mass and respiratory activity. Mitochondrion.

[32] Tin A, Grams ME, Ashar FN, Lane JA, Rosenberg AZ, Grove ML, Boerwinkle E, Selvin E, Coresh J, Pankratz N (2016). Association between mitochondrial DNA copy number in peripheral blood and incident CKD in the atherosclerosis risk in communities study. J Am Soc Nephrol.

[33] Ashar FN, Moes A, Moore AZ, Grove ML, Chaves PHM, Coresh J, Newman AB, Matteini AM, Bandeen-Roche K, Boerwinkle E (2015). Association of mitochondrial DNA levels with frailty and all-cause mortality. J Mol Med (Berl).

[34] O’Hara R, Tedone E, Ludlow A, Huang E, Arosio B, Mari D, Shay JW (2019). Quantitative mitochondrial DNA copy number determination using droplet digital PCR with single-cell resolution. Genome Res.

[35] Butow RA, Avadhani NG (2004). Mitochondrial signaling: the retrograde response. Mol Cell.

[36] Alam TI, Kanki T, Muta T, Ukaji K, Abe Y, Nakayama H, Takio K, Hamasaki N, Kang D (2003). Human mitochondrial DNA is packaged with TFAM. Nucleic Acids Res.

[37] Morozov YI, Parshin AV, Agaronyan K, Cheung ACM, Anikin M, Cramer P, Temiakov D (2015). A model for transcription initiation in human mitochondria. Nucleic Acids Res.

[38] Jeng J-Y, Yeh T-S, Lee J-W, Lin S-H, Fong T-H, Hsieh R-H (2008). Maintenance of mitochondrial DNA copy number and expression are essential for preservation of mitochondrial function and cell growth. J Cell Biochem.

[39] Reznik E, Miller ML, Şenbabaoğlu Y, Riaz N, Sarungbam J, Tickoo SK, Al-Ahmadie HA, Lee W, Seshan VE, Hakimi AA (2016). Mitochondrial DNA copy number variation across human cancers. Elife.

[40] Kopinski PK, Singh LN, Zhang S, Lott MT, Wallace DC (2021). Mitochondrial DNA variation and cancer. Nat Rev Cancer.

[41] Scholte HR (1988). The biochemical basis of mitochondrial diseases. J Bioenerg Biomembr.

[42] Aon MA, Cortassa S, Juhaszova M, Sollott SJ (2016). Mitochondrial health, the epigenome and healthspan. Clin Sci (Lond).

[43] Holt IJ, Reyes A (2012). Human mitochondrial DNA replication. Cold Spring Harb Perspect Biol.

[44] Hudson G, Chinnery PF (2006). Mitochondrial DNA polymerase-γ and human disease. Hum Mol Genet.

[45] Tyynismaa H, Mjosund KP, Wanrooij S, Lappalainen I, Ylikallio E, Jalanko A, Spelbrink JN, Paetau A, Suomalainen A (2005). Mutant mitochondrial helicase Twinkle causes multiple MtDNA deletions and a late-onset mitochondrial disease in mice. Proc Natl Acad Sci.

[46] Mayr JA, Haack TB, Freisinger P, Karall D, Makowski C, Koch J, Feichtinger RG, Zimmermann FA, Rolinski B, Ahting U (2015). Spectrum of combined respiratory chain defects. J Inherit Metab Dis.

[47] Chistiakov DA, Sobenin IA, Revin VV, Orekhov AN, Bobryshev YV (2014). Mitochondrial aging and age-related dysfunction of mitochondria. Biomed Res Int.

[48] Koklesova L, Liskova A, Samec M, Zhai K, Al-Ishaq RK, Bugos O, Šudomová M, Biringer K, Pec M, Adamkov M (2021). Protective effects of flavonoids against mitochondriopathies and associated pathologies: focus on the predictive approach and personalized prevention. Int J Mol Sci.

[49] Sciacco M, Bonilla E, Schon EA, DiMauro S, Moraes CT (1994). Distribution of wild-type and common deletion forms of MtDNA in normal and respiration-deficient muscle fibers from patients with mitochondrial myopathy. Hum Mol Genet.

[50] Chacko BK, Zhi D, Darley-Usmar VM, Mitchell T (2016). The bioenergetic health index is a sensitive measure of oxidative stress in human monocytes. Redox Biol.

[51] Chacko BK, Kramer PA, Ravi S, Benavides GA, Mitchell T, Dranka BP, Ferrick D, Singal AK, Ballinger SW, Bailey SM (2014). The bioenergetic health index: a new concept in mitochondrial translational research. Clin Sci (Lond).

[52] Plitzko B, Loesgen S (2018). Measurement of oxygen consumption rate (OCR) and extracellular acidification rate (ECAR) in culture cells for assessment of the energy metabolism. Bio Protoc.

[53] Birsoy K, Wang T, Chen W, Freinkman E, Abu-Remaileh M, Sabatini DM (2015). An Essential role of the mitochondrial electron transport chain in cell proliferation is to enable aspartate synthesis. Cell.

[54] Pagliarini DJ, Rutter J (2013). Hallmarks of a New Era in Mitochondrial Biochemistry. Genes Dev.

[55] Pfleger J, He M, Abdellatif M (2015). Mitochondrial complex II is a source of the reserve respiratory capacity that is regulated by metabolic sensors and promotes cell survival. Cell Death Dis.

[56] Mitchell P (1961). Coupling of phosphorylation to electron and hydrogen transfer by a chemi-osmotic type of mechanism. Nature.

[57] Dranka BP, Hill BG, Darley-Usmar VM (2010). Mitochondrial reserve capacity in endothelial cells: the impact of nitric oxide and reactive oxygen species. Free Radic Biol Med.

[58] Cheng J, Nanayakkara G, Shao Y, Cueto R, Wang L, Yang WY, Tian Y, Wang H, Yang X (2017). Mitochondrial proton leak plays a critical role in pathogenesis of cardiovascular diseases. Adv Exp Med Biol.

[59] Nolfi-Donegan D, Braganza A, Shiva S (2020). Mitochondrial electron transport chain: oxidative phosphorylation, oxidant production, and methods of measurement. Redox Biol.

[60] Nagaraj R, Sharpley MS, Chi F, Braas D, Zhou Y, Kim R, Clark AT, Banerjee U (2017). Nuclear localization of mitochondrial TCA cycle enzymes as a critical step in mammalian zygotic genome activation. Cell.

[61] Guyatt AL, Burrows K, Guthrie PAI, Ring S, McArdle W, Day INM, Ascione R, Lawlor DA, Gaunt TR, Rodriguez S (2018). Cardiometabolic phenotypes and mitochondrial DNA copy number in two cohorts of UK women. Mitochondrion.

[62] Lee M, Yoon J-H (2015). Metabolic interplay between glycolysis and mitochondrial oxidation: the reverse Warburg effect and its therapeutic implication. World J Biol Chem.

[63] Samec M, Liskova A, Koklesova L, Samuel SM, Zhai K, Buhrmann C, Varghese E, Abotaleb M, Qaradakhi T, Zulli A (2020). Flavonoids against the warburg phenotype-concepts of predictive, preventive and personalised medicine to cut the Gordian knot of cancer cell metabolism. EPMA J.

[64] Zhao H, Shen J, Leung E, Zhang X, Chow W-H, Zhang K (2020). Leukocyte mitochondrial DNA Copy number and built environment in Mexican Americans: a cross-sectional study. Sci Rep.

[65] Moore A, Lan Q, Hofmann JN, Liu C-S, Cheng W-L, Lin T-T, Berndt SI (2017). A Prospective study of mitochondrial DNA copy number and the risk of prostate cancer. Cancer Causes Control.

[66] Yang P-K, Chou C-H, Chang C-H, Chen S-U, Ho H-N, Chen M-J (2020). Changes in peripheral mitochondrial DNA copy number in metformin-treated women with polycystic ovary syndrome: a longitudinal study. Reprod Biol Endocrinol.

[67] Jain A, Bakhshi S, Thakkar H, Gerards M, Singh A. Elevated mitochondrial DNA copy numbers in pediatric acute lymphoblastic leukemia: a potential biomarker for predicting inferior survival. *Pediatr Blood Cancer* **2018**, *65*, 10.1002/pbc.26874. 10.1002/pbc.26874 29134740

[68] Chien MC, Huang WT, Wang PW, Liou CW, Lin TK, Hsieh CJ, Weng SW (2012). Role of mitochondrial DNA variants and copy number in diabetic atherogenesis. Genet Mol Res.

[69] Ridge PG, Maxwell TJ, Foutz SJ, Bailey MH, Corcoran CD, Tschanz JT, Norton MC, Munger RG, O’Brien E, Kerber RA (2014). Mitochondrial genomic variation associated with higher mitochondrial copy number: the Cache County Study on Memory Health and Aging. BMC Bioinformatics.

[70] Kramer PA, Chacko BK, George DJ, Zhi D, Wei C-C, Dell’Italia LJ, Melby SJ, George JF, Darley-Usmar VM (2015). Decreased bioenergetic health index in monocytes isolated from the pericardial fluid and blood of post-operative cardiac surgery patients. Biosci Rep.

[71] Vayalil PK, Landar A (2015). Mitochondrial oncobioenergetic index: a potential biomarker to predict progression from indolent to aggressive prostate cancer. Oncotarget.

[72] Czajka A, Ajaz S, Gnudi L, Parsade CK, Jones P, Reid F, Malik AN (2015). Altered mitochondrial function, mitochondrial DNA and reduced metabolic flexibility in patients with diabetic nephropathy. EBioMedicine.

[73] Bersani FS, Morley C, Lindqvist D, Epel ES, Picard M, Yehuda R, Flory J, Bierer LM, Makotkine I, Abu-Amara D (2016). Mitochondrial DNA copy number is reduced in male combat veterans with PTSD. Prog Neuropsychopharmacol Biol Psychiatry.

[74] Kim M-Y, Lee J-W, Kang H-C, Kim E, Lee D-C (2011). Leukocyte mitochondrial DNA (MtDNA) Content is associated with depression in old women. Arch Gerontol Geriatr.

[75] Filograna R, Koolmeister C, Upadhyay M, Pajak A, Clemente P, Wibom R, Simard ML, Wredenberg A, Freyer C, Stewart JB (2019). Modulation of MtDNA copy number ameliorates the pathological consequences of a heteroplasmic MtDNA mutation in the mouse. Sci Adv.

[76] Ikeda M, Ide T, Fujino T, Arai S, Saku K, Kakino T, Tyynismaa H, Yamasaki T, Yamada K-I, Kang D (2015). Overexpression of TFAM or twinkle increases MtDNA copy number and facilitates cardioprotection associated with limited mitochondrial oxidative stress. PLoS ONE.

[77] Xu X, Jin K, Bais AS, Zhu W, Yagi H, Feinstein TN, Nguyen PK, Criscione JD, Liu X, Beutner G (2022). Uncompensated mitochondrial oxidative stress underlies heart failure in an IPSC-derived model of congenital heart disease. Cell Stem Cell.

[78] Kakinuma Y, Miyauchi T, Yuki K, Murakoshi N, Goto K, Yamaguchi I (2000). Mitochondrial dysfunction of cardiomyocytes causing impairment of cellular energy metabolism induces apoptosis, and concomitant increase in cardiac endothelin-1 expression. J Cardiovasc Pharmacol.

[79] Mondal NK, Li T, Chen Z, Chen HH, Sorensen EN, Pham SM, Sobieski MA, Koenig SC, Slaughter MS, Griffith BP (2017). Mechanistic insight of platelet apoptosis leading to non-surgical bleeding among heart failure patients supported by continuous-flow left ventricular assist devices. Mol Cell Biochem.

[80] McCutcheon K, Dickens C, van Pelt J, Dix-Peek T, Grinter S, McCutcheon L, Patel A, Hale M, Tsabedze N, Vachiat A (2019). Dynamic changes in the molecular signature of adverse left ventricular remodeling in patients with compensated and decompensated chronic primary mitral regurgitation. Circ Heart Fail.

[81] Zhu H, Tan Y, Du W, Li Y, Toan S, Mui D, Tian F, Zhou H (2021). Phosphoglycerate mutase 5 exacerbates cardiac ischemia-reperfusion injury through disrupting mitochondrial quality control. Redox Biol.

[82] Hortmann M, Robinson S, Mohr M, Mauler M, Stallmann D, Reinöhl J, Duerschmied D, Peter K, Carr J, Gibson CM (2019). The mitochondria-targeting peptide elamipretide diminishes circulating HtrA2 in ST-segment elevation myocardial infarction. Eur Heart J Acute Cardiovasc Care.

[83] Smenes BT, Bækkerud FH, Slagsvold KH, Hassel E, Wohlwend M, Pinho M, Høydal M, Wisløff U, Rognmo Ø, Wahba A (2018). Acute exercise is not cardioprotective and may induce apoptotic signalling in heart surgery: a randomized controlled trial. Interact Cardiovasc Thorac Surg.

[84] Xu Y, Guo W, Zeng D, Fang Y, Wang R, Guo D, Qi B, Xue Y, Xue F, Jin Z (2021). Inhibiting MiR-205 alleviates cardiac ischemia/reperfusion injury by regulating oxidative stress, mitochondrial function, and apoptosis. Oxid Med Cell Longev.

[85] Gong L, Tang Y, An R, Lin M, Chen L, Du J (2017). RTN1-C mediates cerebral ischemia/reperfusion injury via ER stress and mitochondria-associated apoptosis pathways. Cell Death Dis.

[86] Isidoro A, Martínez M, Fernández PL, Ortega AD, Santamaría G, Chamorro M, Reed JC, Cuezva JM (2004). Alteration of the bioenergetic phenotype of mitochondria is a hallmark of breast, gastric, lung and oesophageal cancer. Biochem J.

[87] Mitov MI, Harris JW, Alstott MC, Zaytseva YY, Evers BM, Butterfield DA (2017). Temperature induces significant changes in both glycolytic reserve and mitochondrial spare respiratory capacity in colorectal cancer cell lines. Exp Cell Res.

[88] Stasi C, Fallani S, Voller F, Silvestri C (2020). Treatment for COVID-19: An Overview. Eur J Pharmacol.

[89] Gibellini L, De Biasi S, Paolini A, Borella R, Boraldi F, Mattioli M, Lo Tartaro D, Fidanza L, Caro-Maldonado A, Meschiari M (2020). Altered bioenergetics and mitochondrial dysfunction of monocytes in patients with COVID-19 pneumonia. EMBO Mol Med.

[90] Yan X, Hu Y, Wang B, Wang S, Zhang X (2020). Metabolic dysregulation contributes to the progression of Alzheimer’s disease. Front Neurosci.

[91] Dematteis G, Vydmantaitė G, Ruffinatti FA, Chahin M, Farruggio S, Barberis E, Ferrari E, Marengo E, Distasi C, Morkūnienė R (2020). Proteomic analysis links alterations of bioenergetics, mitochondria-ER interactions and proteostasis in hippocampal astrocytes from 3xTg-AD mice. Cell Death Dis.

[92] Xu Y, Cheng L, Sun J, Li F, Liu X, Wei Y, Han M, Zhu Z, Bi J, Lai C (2021). Hypermethylation of mitochondrial cytochrome b and cytochrome c oxidase II genes with decreased mitochondrial DNA copy numbers in the APP/PS1 transgenic mouse model of Alzheimer’s disease. Neurochem Res.

[93] Saran R, Li Y, Robinson B, Ayanian J, Balkrishnan R, Bragg-Gresham J, Chen JTL, Cope E, Gipson D, He K (2015). US Renal Data System 2014 Annual Data Report: epidemiology of Kidney Disease in the United States. Am J Kidney Dis.

[94] Eirin A, Saad A, Tang H, Herrmann SM, Woollard JR, Lerman A, Textor SC, Lerman LO (2016). Urinary mitochondrial DNA copy number identifies chronic renal injury in hypertensive patients. Hypertension.

[95] Jiang H, Shao X, Jia S, Qu L, Weng C, Shen X, Wang Y, Huang H, Wang Y, Wang C (2019). The Mitochondria-targeted metabolic tubular injury in diabetic kidney disease. Cell Physiol Biochem.

[96] Chelimsky G, Simpson P, Zhang L, Bierer D, Komas S, Kalyanaraman B, Chelimsky T (2021). Impaired mitochondrial bioenergetics function in pediatric chronic overlapping pain conditions with functional gastrointestinal disorders. Pain Res Manag.

[97] Lee H, Oh S, Yang W, Park R, Kim H, Jeon JS, Noh H, Han DC, Cho KW, Kim YJ (2019). Bariatric surgery reduces elevated urinary mitochondrial DNA copy number in patients with obesity. J Clin Endocrinol Metab.

[98] Chacko B, Culp ML, Bloomer J, Phillips J, Kuo Y-F, Darley-Usmar V, Singal AK (2019). Feasibility of cellular bioenergetics as a biomarker in porphyria patients. Mol Genet Metab Rep.

[99] Smolen JS, Aletaha D, McInnes IB (2016). Rheumatoid Arthritis. Lancet.

[100] Gautam S, Kumar U, Kumar M, Rana D, Dada R (2021). Yoga improves mitochondrial health and reduces severity of autoimmune inflammatory arthritis: a randomized controlled trial. Mitochondrion.

[101] Wang L, Wang D, Zou X, Xu C (2009). Mitochondrial Functions on Oocytes and Preimplantation Embryos. J Zhejiang Univ Sci B.

[102] Yu Y, Dumollard R, Rossbach A, Lai FA, Swann K (2010). Redistribution of mitochondria leads to bursts of ATP production during spontaneous mouse oocyte maturation. J Cell Physiol.

[103] Labarta E, de Los Santos MJ, Escribá MJ, Pellicer A, Herraiz S (2019). Mitochondria as a Tool for Oocyte Rejuvenation. Fertil Steril.

[104] Amoushahi M, Salehnia M, Mowla SJ (2017). Vitrification of mouse MII oocyte decreases the mitochondrial DNA copy number, TFAM gene expression and mitochondrial enzyme activity. J Reprod Infertil.

[105] Koklesova L, Liskova A, Samec M, Qaradakhi T, Zulli A, Smejkal K, Kajo K, Jakubikova J, Behzadi P, Pec M, et al. Genoprotective activities of plant natural substances in cancer and chemopreventive strategies in the context of 3P medicine. EPMA J. 2020;11:261–87. 10.1007/s13167-020-00210-5. 10.1007/s13167-020-00210-5 PMC7272522 32547652

[106] Polivka J, Polivka J, Pesta M, Rohan V, Celedova L, Mahajani S, Topolcan O, Golubnitschaja O (2019). Risks associated with the stroke predisposition at young age: facts and hypotheses in light of individualized predictive and preventive approach. EPMA J.

[107] *Flammer Syndrome: From Phenotype to Associated Pathologies, Prediction, Prevention and Personalisation*; Golubnitschaja, O., Ed.; Advances in predictive, preventive and personalised medicine; Springer International Publishing, 2019; ISBN 978–3–030–13549–2.

